# Identification of key genes for heart failure in dilated cardiomyopathy in different populations

**DOI:** 10.3389/fgene.2025.1618390

**Published:** 2025-10-14

**Authors:** Yue Yu, Chentian Xue, Dong Ji, Wei Sheng, Xiang Gao, Xize Wu, Chengyan Wu

**Affiliations:** ^1^ Nantong Hospital of Traditional Chinese Medicine, Nantong Hospital Affiliated to Nanjing University of Chinese Medicine, Nantong, Jiangsu, China; ^2^ Nanjing University of Chinese Medicine, Nanjing, Jiangsu, China; ^3^ Liaoning University of Traditional Chinese Medicine, Shenyang, China

**Keywords:** dilated cardiomyopathy, heart failure, bioinformatics, machine learning models, genes

## Abstract

**Background:**

Heart failure (HF) represents the end stage of cardiovascular disease and is the leading cause of mortality. The objective of this study was to identify potential biomarkers and elucidate the mechanisms underlying the development of HF across diverse populations and among different genders.

**Methods:**

This study strictly included five datasets of HF with dilated cardiomyopathy: GSE141910 (African American and Caucasian), GSE57345 (USA), GSE21610 (Germany), GSE17800 (Germany), and GSE42955 (Spain). These datasets were merged and normalized as the validation set. Differentially expressed genes (DEGs) were identified through differential expression analysis, and module genes were identified using weighted gene co-expression network analysis. Subsequent stratification by gender and ethnicity (African American, Caucasian, German, and Spanish) was performed, followed by immune infiltration analysis. Finally, the least absolute shrinkage and selection operator (LASSO) regression, support vector machine-recursive feature elimination (SVM-REF), and random forest (RF) models were used to screen for Hub genes and to construct a nomogram predicting the occurrence of HF in different populations based on these Hub genes. Additionally, GSE3585, GSE120895, GSE5406, and GSE1145 serve as the validation set.

**Results:**

A total of 650 samples were included (323 controls and 327 HF samples), including 122 African American samples (44 controls and 78 HF samples), 238 Caucasian samples (122 controls and 116 HF samples), 55 German samples (16 controls and 39 HF samples), and 17 Spanish samples (5 controls and 12 HF samples). Functional enrichment analysis demonstrated that the pathogenesis of HF is closely related to the inflammatory response, immune response, vascular regulation, the Wnt signaling pathway, glutathione metabolism, sphingolipid metabolism, and apoptosis. Immune infiltration analysis showed that HF patients exhibited a high abundance of resting mast cells, resting NK cells, CD8T cells, resting memory CD4 T cells, activated memory CD4 T cells, M1 Macrophages, naive CD4 T cells, M0 Macrophages, regulatory T cells (Tregs), follicular helper T cells, Monocytes, and activated NK cells, and a lower abundance of plasma cells, neutrophils, and eosinophils. Multiple machine learning analyses identified MYH6, ASPN, and COL14A1 as Hub genes, NAP1L3, PLEKHH2, MOXD1, CCDC80, CA14, and SERPINE2 as male-specific, CX3CR1, SYN2, and SLC25A18 as female-specific, and NQO1, KAZALD1, and UBASH3A as African American male-specific, SYN2 as African American female-specific, CD83, C1QTNF3, GRB14, and MOXD1 as Caucasian male-specific, CD83, VIT, and PODXL2 as Caucasian female-specific, LSAMP and C14orf132 as German male-specific, and LSAMP and BMP4 as German female-specific, CIART and SNORA80E as Spanish-specific DEGs. Hub genes are strongly associated with M1 macrophages.

**Conclusion:**

The biomarkers of HF vary significantly across different populations and genders. MYH6, ASPN, and COL14A1 may be potential biomarkers for HF in dilated cardiomyopathy.

## 1 Introduction

Heart failure (HF) is a clinical syndrome recognized as a global epidemic, representing the end stage of most cardiovascular diseases, and is one of the leading causes of death and disability. Over the past few decades, the incidence of HF has gradually reached a stable and decreasing trend in developed countries. For instance, the incidence rates are 6.5 per 1,000 in Germany, 2.9–3.9 per 1,000 in Spain, 2.2–3.2 per 1,000 in America, and 0.7 per 1,000 in Hong Kong (Conrad et al., 2018; Savarese et al., 2023). Despite this slight decline in incidence, the prevalence of HF is gradually increasing and varies considerably across countries and regions. The highest prevalence in 2017 was noted in Central Europe, North Africa, and the Middle East, while lower rates were observed in Eastern Europe and Southeast Asia. The prevalence rates range from 0.9% to 6.8% in Spain, 3.9% in Germany, 2.4%–3.0% in America, and 0.4% in Thailand (Savarese et al., 2023). Furthermore, HF imposes a significant economic burden on healthcare systems worldwide, with the total cost of treating HF in the United States projected to rise from $31 billion to $70 billion between 2012 and 2030 (Savarese and Lund, 2017; Heidenreich et al., 2013). This escalation undoubtedly places a substantial strain on healthcare expenditures.

The diagnosis of HF primarily relies on electrocardiograms, imaging, laboratory tests, and biomarker assessments. B-type Natriuretic Peptide (BNP) and N-terminal Pro-B-Type Natriuretic Peptide (NT-proBNP) are widely regarded as the most effective biomarkers for diagnosing HF due to their significant roles in diagnosis and prognostic evaluation (Castiglione et al., 2022). The American College of Cardiology/American Heart Association (ACC/AHA) endorses the use of BNP and NT-proBNP to assist in diagnosing HF (Yancy et al., 2017). However, the European Society of Cardiology (ESC) guidelines advocate for the use of these biomarkers to rule out HF, considering the impact of gender, age, and comorbidities (Ponikowski et al., 2016). Consequently, many guidelines suggest that thresholds for biomarkers should be determined with consideration for age, gender, and ethnic region stratification (Kavsak et al., 2019). In terms of treatment, HF therapeutic strategies have evolved from the traditional “Golden Triangle”- comprising angiotensin-converting enzyme inhibitors or angiotensin II receptor antagonists, beta-blockers, and mineralocorticoid receptor antagonists-to the “New Quadruple Combination,” which adds sodium-glucose cotransporter 2 inhibitors (McDonagh et al., 2021). The approach has further advanced to the current “Five Golden Flowers,” with the addition of soluble guanylate cyclase stimulators, such as vericiguat (Metra et al., 2023). This progression is due to the residual risk of HF exacerbation and death that persists even with the “New Quadruple Combination” therapy (Docherty et al., 2020). It highlights that, despite ongoing advancements in HF drug development, the diagnosis and treatment of HF continue to face numerous challenges. These challenges stem from the complexity and refractory nature of HF, as well as its poor prognosis, and include individual differences, comorbidity management, medication side effects, and economic burdens.

Significant gender differences exist in HF, encompassing symptoms, susceptibility, risk factors, pathophysiology, and response to treatment. Studies have shown that women are more likely to present with severe symptoms, with dyspnea being more predominant in women and peripheral edema in men (Maidana et al., 2023). Risk factors such as hypertension, diabetes, smoking, and obesity make women more susceptible to HF than men. Additionally, women face unique risk factors, including those related to menopause, breast cancer treatments, and pregnancy (Maidana et al., 2023; Lala et al., 2022). Biomarkers, including NT-proBNP, CA125, high-sensitivity troponin, galectin-3, and osteopontin, also exhibit gender-specific differences (Maidana et al., 2023). Racial differences are equally important factors influencing the diagnosis and prognosis of HF. Research published in the Journal of the American Medical Association indicates that African American individuals have nearly twice the incidence of HF, experience an earlier onset of the disease, present with higher severity at diagnosis, and have a higher mortality rate among younger individuals (45–64 years) (Yancy, 2024). Age is also a key risk factor for HF, with significant variations in incidence, progression, and biomarker expression across different age groups. Global Burden of Disease database analysis indicates that the number of HF cases increases significantly with age, particularly among individuals over 65 years old (Ran et al., 2025; Kang et al., 2025). Multiple studies have demonstrated that NT-proBNP levels, a marker for HF, exhibit significant variations across different age groups and genders, with elevated levels being more common in the general middle-aged population (Welsh et al., 2022; Mu et al., 2023). Therefore, studies focusing on gender, age, and racial differences may offer insights to improve the diagnosis of HF and potentially contribute to the development of new drugs targeting HF.

Due to database limitations, specifically the lack of basic age information, this study focused on four distinct populations: African American, Caucasian, German, and Spain. Differential expression analysis was employed to identify gender-specific differentially expressed genes (DEGs) within these populations. Enrichment analysis was utilized to explore the potential pathogenesis of HF, while immune infiltration analysis was applied to investigate the microenvironment of immune infiltration across populations. Additionally, Weighted Gene Co-expression Network Analysis (WGCNA) was applied to identify the most relevant gene modules associated with HF in different populations. Finally, three machine learning models were applied to screen for gender-specific Hub genes in different populations: the least absolute shrinkage and selection operator (LASSO) regression, support vector machine-recursive feature elimination (SVM-REF), and random forest (RF). These models were used to construct nomograms to predict the risk of HF.

## 2 Materials and methods

### 2.1 Dataset acquisition

Five HF-related datasets were obtained from the Gene Expression Omnibus database (GEO, https://www.ncbi.nlm.nih.gov/geo/) (Clough and Barrett, 2016), including the GSE141910 (Tan et al., 2020) and GSE57345 (Jia et al., 2015) databases from the America, the GSE21610 (Schwientek et al., 2010) and GSE17800 (Ameling et al., 2013) databases from the Germany, and the GSE42955 (Molina-Navarro et al., 2013) from the Spain. In addition, the German related datasets GSE3585 (Barth et al., 2006) and GSE120895 (Witt et al., 2019) and the American related datasets GSE5406 (Hannenhalli et al., 2006) and GSE1145 were used for external validation (Table 1). This study, the validation cohort strictly included HF samples with dilated cardiomyopathy and excluded those with HF secondary to ischemic cardiomyopathy. Regarding ethnicity and population issues, the GSE141910 dataset provided detailed information on population ethnicity (African American and Caucasian). The GSE21610 and GSE17800 datasets were sourced from Germany, and the GSE42955 dataset was from Spain.

**TABLE 1:** Dataset information of dilated cardiomyopathy heart failure.

Role	Dataset	Platform	Race	Source	Disease	Sample size	Sample information
Training Set	GSE141910	GPL16791	United States (African American and Caucasian)	Left Ventricle	Dilated Cardiomyopathy	360	122 African American 44 control (23 female and 21 male) 78 HF (37 female and 41 male) 238 Caucasian 122 control (66 female and 56 male) 116 HF (40 female and 76 male)
Training Set	GSE57345	GPL9052	United States	Left Ventricle	Idiopathic Dilated Cardiomyopathy	218	136 control (63 female and 73 male) 82 HF (19 female and 63 male)
Training Set	GSE21610	GPL570	Germany	Left Ventricle	Dilated Cardiomyopathy	29	8 control (2 female and 6 male) 21 HF (2 female and 19 male)
Training Set	GSE17800	GPL570	Germany	Endocardial Myocardium	Dilated Cardiomyopathy	26	8 control (2 female and 6 male) 18 HF (5 female and 13 male)
Training Set	GSE42955	GPL6244	Spain	Left Ventricle	Dilated Cardiomyopathy	17	5 control (0 female and 5 male) 12 HF (0 female and 12 male)
Validation Set	GSE3585	GPL96	Germany	Left Ventricle	Dilated Cardiomyopathy	12	5 control and 7 HF.
Validation Set	GSE120895	GPL570	Germany	Endocardial Myocardium	Dilated Cardiomyopathy	55	8 control and 47 HF.
Validation Set	GSE5406	GPL96	United States	Myocardium	Dilated Cardiomyopathy	102	18 control and 86 HF.
Validation Set	GSE1145	GPL570	United States	Left Ventricle	Dilated Cardiomyopathy	55	37 control and 18 HF.

### 2.2 Identification of DEGs

In this study, different datasets were merged and normalized using the “Affy” R package (Gautier et al., 2004). To address batch effects arising from different platforms and studies, cross-platform batch effect correction was performed using the ComBat algorithm from the “SVA” R package (Leek et al., 2012). A model matrix incorporating the biological condition (disease vs control) was included as a covariate to preserve biological variance while removing technical artifacts. The effectiveness of batch correction was visually assessed through both Principal Component Analysis (PCA) and Uniform Manifold Approximation and Projection (UMAP) plots before and after correction. DEGs were identified using the “limma” R package with a linear modeling approach with a threshold of |log_2_ fold change| >1 (2-fold differential expression) and *P* < 0.05 (Ritchie et al., 2015).

### 2.3 Functional enrichment analysis and construction of protein-protein interaction network

The genes were imported into the DAVID database (https://david.ncifcrf.gov/home.jsp) for analysis of Biological Process, Cellular Component, Molecular Function, and Pathway. Subsequently, the genes were imported into the STRING database (https://cn.string-db.org/) to construct protein-protein interaction networks.

### 2.4 Gene set enrichment analysis (GSEA)

The “GSEA” R package is used to calculate the correlation between Hub genes and other genes, and then all genes are ranked according to the correlation from the highest to the lowest, and the enriched set of genes at the bottom of the ranking is detected and analyzed (Subramanian et al., 2005).

### 2.5 Immune infiltration analysis and correlation analysis

The relative abundance of 22 immune cell types was estimated using the CIBERSORT deconvolution algorithm with the LM22 signature matrix (Chen et al., 2018). To ensure robust results, the number of permutations was set to 1000 and quantile normalization (QN = TRUE) was applied to the input data. Wilcoxon rank-sum tests were used for intergroup comparisons, with FDR correction applied using the Benjamini-Hochberg method. Subsequently, Spearman correlation analysis was employed to reveal the relationship between Hub genes and immune cells.

### 2.6 WGCNA

The “WGCNA” R package is used to remove the outlier samples and construct a co-expression network of gene expression matrices for the remaining samples. The soft threshold corresponding to fit R^2^ = 0.8 was chosen for the construction of gene modules, while the minimum number of module genes (minSize) was specified to be 10, and the most relevant module for the trait was selected (Langfelder and Horvath, 2008).

### 2.7 Single-gene analysis of variance

Candidate Hub genes were further screened for Hub genes in the external validation set using the Wilcoxon rank-sum test for single-gene difference analysis.

### 2.8 Machine learning models

The “glmnet” “e1071” “kernlab” “caret” “randomForest” R packages R packages were used to establish machine learning models screening for the Hub genes, including the LASSO regression, SVM-REF, and RF model (Engebretsen and Bohlin, 2019; Van Essen, 2012). The area under the receiver operating characteristic (ROC) curve was visualized using the “pROC” R package (Robin et al., 2011).

### 2.9 Construction and validation of a nomogram model

A nomogram model was established using the “rms” R package to predict the probability of the occurrence of AS, and its predictive power was estimated by using calibration curves and decision curve analysis.

### 2.10 Gene-drug/chemical interaction

DGIdb 5.0 integrates drug-gene interactions from multiple databases including DrugBank, Drug Target Commons, and TTD. Its core methodology involves associative analysis of drugs and genes, establishing an interaction network through the integration of public data and experimental validation (Cannon et al., 2024). However, its predictive results contain numerous correlations that have not been experimentally verified and should be treated with caution. CoreMine Database is a literature-based precision data mining service platform. The Hub genes were imported into the DGIdb database (https://dgidb.org/) and CoreMine database (https://coremine.com/medical/#search) to predict the corresponding Drug, Chemical, and Food.

## 3 Results

### 3.1 Identification of DEGs in different HF populations

**FIGURE 1:**
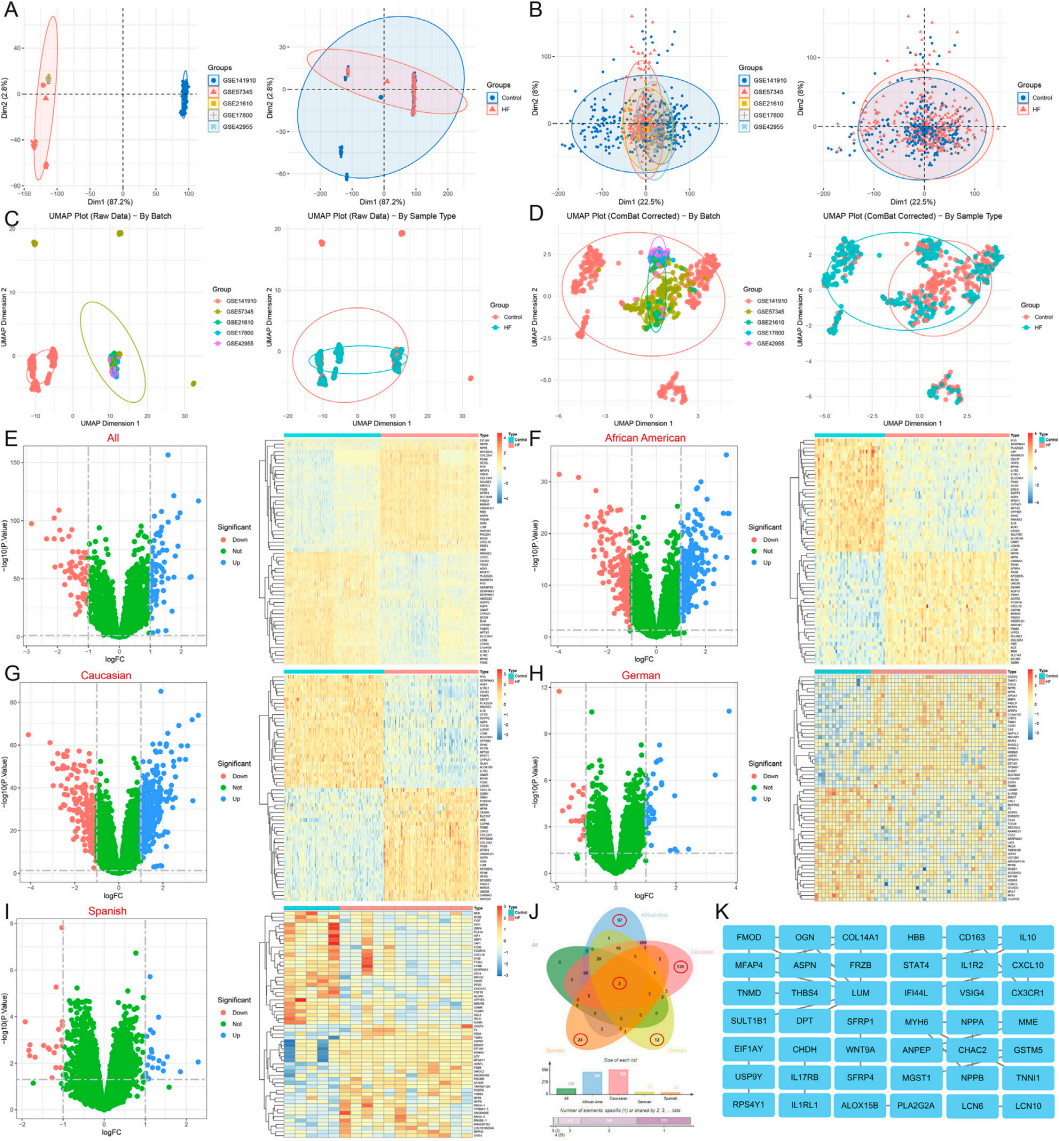
Identification and functional enrichment analysis of DEGs in HF. **(A)** The principal component analysis of the five datasets and clinical characteristics. **(B)** The principal component analysis of the combined dataset and clinical characteristics. **(C)** The UMAP of the five datasets and clinical characteristics. **(D)** The UMAP of the combined dataset and clinical characteristics. **(E–I)** Volcano map and heatmap for differential expression analyses of **(E)** all, **(F)** African American, **(G)** Caucasian, **(H)** German, and **(I)** Spanish samples. **(J)** The Venn diagram shows 2 common DEGs and 97 African American-specific, 139 Caucasian-specific, 12 German-specific, and 24 Spanish-specific DEGs. **(K)** Protein-protein interaction network for all sample DEGs.

After merging and standardizing the five datasets to eliminate batch effects, evaluate the merging effect using PCA and UMAP plots (Figures 1A–D). Differential expression analyses were first conducted on a total of 650 samples, comprising 323 normal and 327 HF samples, resulting in the identification of 129 DEGs (Figure 1E). Subsequently, differential expression was analyzed separately for samples from different regions: among African American samples, there were 122 (44 normal and 78 HF), yielding 506 DEGs (Figure 1F); amongst American Caucasian samples, there were 238 (122 normal and 116 HF), yielding 556 DEGs (Figure 1G); amongst German samples, there were 55 (16 normal and 39 HF), yielding 55 DEGs (Figure 1H); amongst Spanish samples, there were 17 (5 normal and 12 HF), yielding 41 DEGs (Figure 1I). A Venn diagram analysis identified 2 common DEGs among all populations (NPPB, STAT4), as well as 97 African American-specific, 139 American Caucasian-specific, 12 German-specific, and 24 Spanish-specific DEGs (Figures 1J,K) (Supplementary Material 1).

**FIGURE 2:**
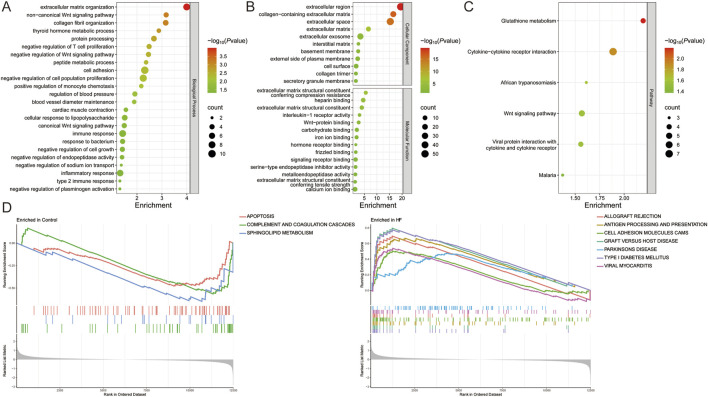
Functional enrichment analysis of DEGs in HF. **(A,B)** The **(A)** biological process, **(B)** cellular component, molecular function, **(C)** pathway, and **(D)** GESA for all sample DEGs.

Functional enrichment of 129 DEGs from the all sample suggests that the biological processes of HF are related to inflammatory response (interleukin-1 receptor activity, positive regulation of monocyte chemotaxis, response to bacterium, cellular response to lipopolysaccharide), immune response (negative regulation of T cell proliferation, type 2 immune response), vascular regulation (regulation of blood pressure, blood vessel diameter maintenance, cardiac muscle contraction), the Wnt signaling pathway (Wnt-protein binding, negative regulation of Wnt signaling pathway, canonical and non-canonical Wnt signaling pathway), cell adhesion, migration, and proliferation (extracellular matrix organization, collagen fibril organization, cell adhesion, negative regulation of cell population proliferation, negative regulation of cell growth), metabolic process (thyroid hormone metabolic process, protein processing, peptide metabolic process, negative regulation of endopeptidase activity, metalloendopeptidase activity), ion binding and transfer (negative regulation of sodium ion transport, iron ion binding, calcium ion binding), and are also closely related to glutathione metabolism, cytokine-cytokine receptor interaction, and the Wnt signaling pathway (Figures 2A–C). GSEA also highlights the role of type I diabetes mellitus, antigen processing and presentation, cell adhesion molecules, complement and coagulation cascades, viral myocarditis, Parkinson’s disease, sphingolipid metabolism, and apoptosis in HF (Figure 2D) (Supplementary Material 2).

**FIGURE 3:**
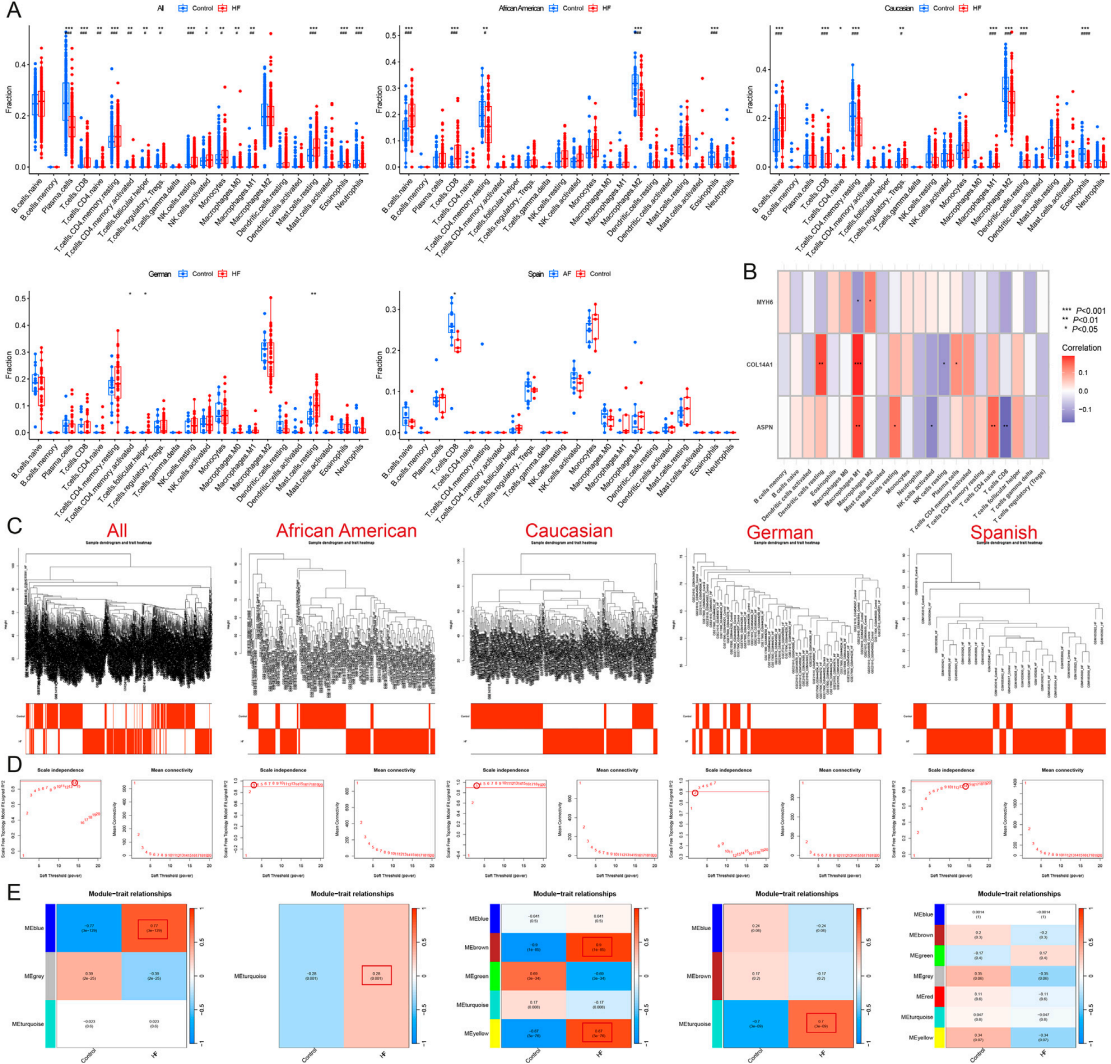
Identification of module genes and immune infiltration analysis in different populations. **(A)** Immune infiltration analysis of all, African American, Caucasian, German, and Spanish samples. **(B)** Correlation analysis of MYH6, ASPN, and COL14A1 with immune cells. **(C)** Sample clustering plot after removal of outlier samples. **(D)** Selection of soft thresholds. **(E)** Gene module of the most relevant genes to HF traits. *P < 0.05, **P < 0.01, ***P < 0.001.

Immune infiltration analysis revealed that HF patients exhibited a higher abundance of resting mast cells, resting NK cells, CD8T cells, resting memory CD4 T cells, activated memory CD4 T cells, M1 Macrophages, naive CD4 T cells, M0 Macrophages, regulatory T cells (Tregs), follicular helper T cells, Monocytes, and activated NK cells, and a lower abundance of plasma cells, neutrophils, and eosinophils (*P* < 0.05 and FDR<0.05). Among African Americans, HF patients had a higher abundance of naïve B cells and CD8 T cells, and a lower abundance of resting memory CD4 T cells, M2 macrophages, and eosinophils (*P* < 0.05 and FDR<0.05). In Caucasians, HF patients showed a higher abundance of naïve CD4 T cells (*P* < 0.05 and FDR>0.05), naïve B cells, CD8 T cells, regulatory T cells, M1 macrophages, and resting dendritic cells, and a lower abundance of resting memory CD4 T cells, M2 macrophages, and eosinophils (*P* < 0.05 and FDR<0.05). In the German population, HF patients demonstrated a higher abundance of resting mast cells and follicular helper T cells, and a lower abundance of resting memory CD4 T cells (*P* < 0.05 and FDR>0.05). In contrast, there were no significant differences in immune cell infiltration observed in the Spanish population (*P* > 0.05) (Figures 3A,B) (Supplementary Material 3). These results suggest that HF patients from different populations have distinct immune infiltration microenvironments.

### 3.2 Identification of candidate hub genes in different HF populations

WGCNA was conducted on these samples to identify the gene modules most strongly associated with HF. When the soft threshold was set to 14, the blue module (*r* = 0.77) was significantly and positively associated with HF across all samples, comprising 200 genes. For African American samples, when the soft threshold was set to 3, the turquoise module (*r* = 0.28) showed a significant positive association with HF and included a total of 3138 genes. At the same soft threshold, both the brown module (*r* = 0.9) and the yellow module (*r* = 0.87) were significantly positively associated with HF in American Caucasian samples, together containing 740 genes. In German samples, when the soft threshold was set to 2, the turquoise module (*r* = 0.7) was significantly positively associated with HF and contained 2099 genes. In Spanish samples, when the soft threshold was set to 14, 7 modules were produced, but none of the modules most relevant to HF (Figures 3C–E).

**FIGURE 4:**
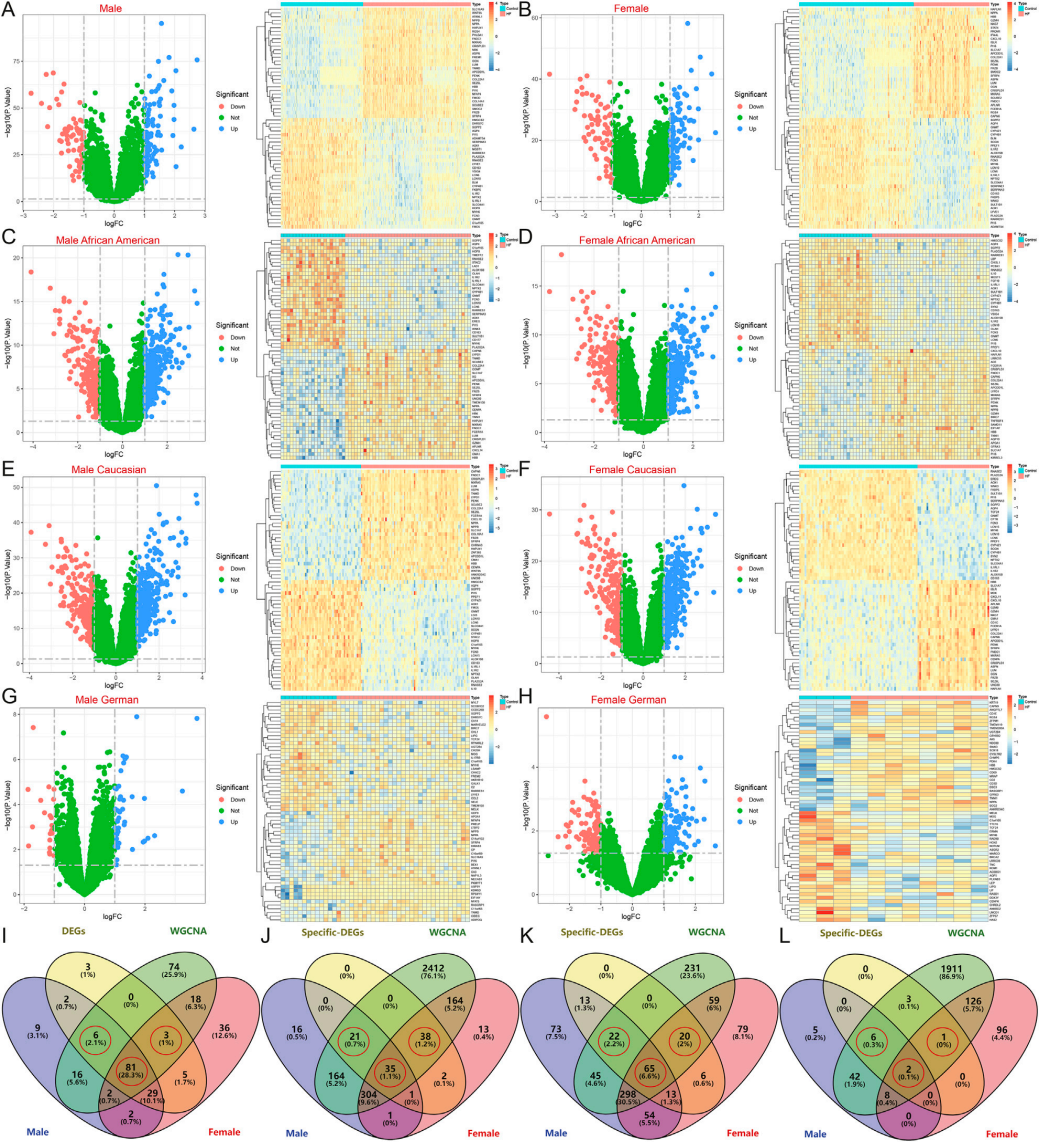
Identification of DEGs after gender stratification in different populations. **(A–H)** Volcano map and heatmap for differential expression analyses of **(A)** all males, **(B)** all females, **(C)** African American males, **(D)** African American females, **(E)** Caucasian males, **(F)** Caucasian females, **(G)** German males, and **(H)** German females. **(I–L)** The Venn diagram shows gender-specific DEGs of **(I)** all samples, **(J)** African Americans, **(K)** Caucasians, and **(L)** Germans.

The samples were then analyzed for differential expression after gender stratification. A total of 374 male samples, comprising 162 normal and 212 HF samples, yielded 147 DEGs (Figure 4A); 259 female samples, including 156 normal and 163 HF samples, yielded 176 DEGs (Figure 4B). Among African American males, 62 samples (21 normal and 41 HF) yielded 542 DEGs (Figure 4C); among African American females, 60 samples (23 normal and 37 HF) yielded 558 DEGs (Figure 4D). Among American Caucasian males, 132 samples (56 normal and 76 HF) yielded 583 DEGs (Figure 4E); among American Caucasian females, 106 samples (66 normal and 40 HF) yielded 594 DEGs (Figure 4F). Among German males, 45 samples (13 normal and 32 HF) yielded 63 DEGs (Figure 4G); among German females, 10 samples (3 normal and 7 HF) yielded 233 DEGs (Figure 4H).

After intersecting with region-specific DEGs, the Venn diagram revealed 81 HF candidate Hub genes, along with 6 male-specific and 3 female-specific modular DEGs (Figure 4I). In the African American samples, there were 35 specific DEGs and 21 male-specific and 38 female-specific modular DEGs (Figure 4J). In the American Caucasian samples, there were 65 specific DEGs and 22 male-specific and 20 female-specific modular DEGs (Figure 4K). In the German samples, there were 2 specific DEGs and 6 male-specific and 1 female-specific modular DEGs (Figure 4L).

**FIGURE 5:**
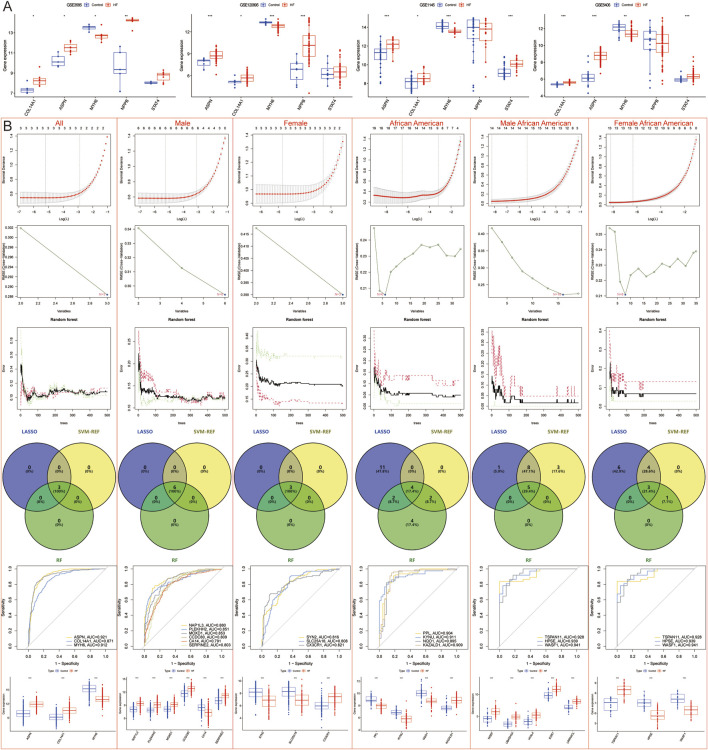
Machine learning models to screen candidate Hub genes. **(A)** External validation of 81 candidate Hub genes in GSE3585, GSE120895, GSE1145, and GSE5406 datasets. **(B)** LASSO, SVM-REF, and RF Screening for Candidate Hub Genes in All, all male, all female, African American, African American male, and African American female samples.

To enhance the screening of candidate Hub genes, external validation was performed in the German datasets GSE3585 and GSE120895, as well as the American datasets GSE5406 and GSE1145. Initially, 81 common HF candidate Hub genes were analyzed by single-gene differential analysis across these datasets, which led to the identification of three DEGs: ASPN, COL14A1, and MYH6, of which ASPN and COL14A1 were upregulated in HF, while MYH6 was downregulated (Figure 5A). Subsequently, to further validate the differential expression of region-specific DEGs. Specifically, American- and Spain-specific DEGs were validated in the German dataset, and German- and Spain-specific DEGs were validated in the American dataset, and genes with differential expression were removed. Ultimately, 33 population-specific, 19 male-specific, and 35 female-specific DEGs were identified in the African American population; 54 population-specific, 19 male-specific, and 17 female-specific DEGs were identified in the American Caucasian population; 1 population-specific, 5 male-specific, and 1 female-specific DEG in the German population; and 20 Spain-specific DEGs were identified.

Correlation analysis of immune infiltration showed that ASPN and COL14A1 were significantly positively correlated with M1 macrophages, whereas MYH6 was negatively correlated with M0 macrophages (Figure 3B).

### 3.3 Machine learning models screening for hub genes

**FIGURE 6:**
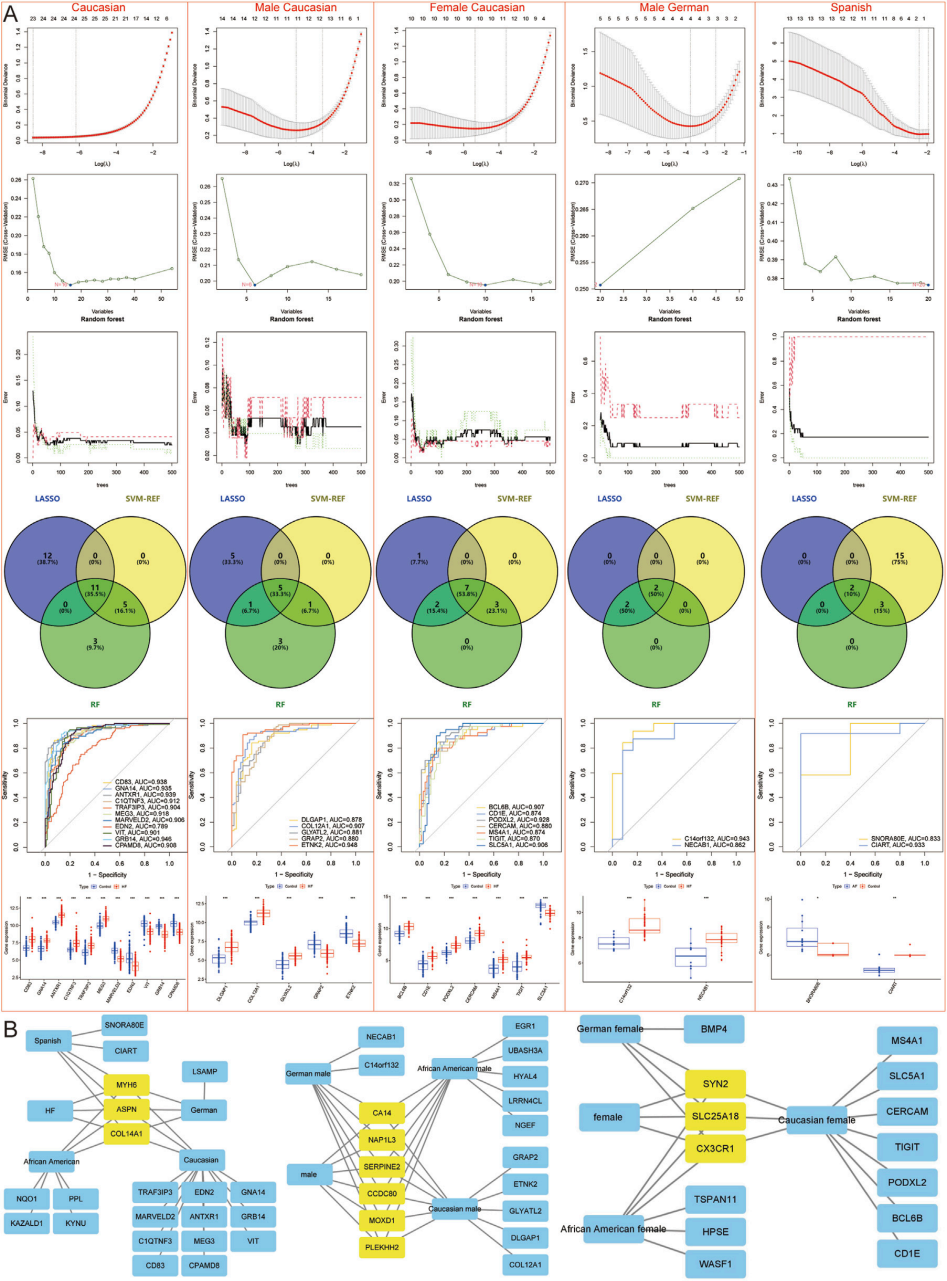
Machine learning models to screen candidate Hub genes. **(A)** LASSO, SVM-REF, and RF Screening for Candidate Hub Genes in Caucasian, Caucasian male, Caucasian female, German male, and Spanish samples. **(B)** Network diagram shows candidate Hub genes.

LASSO regression, SVM-REF, and RF algorithms were employed to further screen for Hub genes and mitigate the risk of overfitting. The results indicated that across all samples, male samples, female samples, African American samples, African American male samples, African American female samples, Caucasian samples, Caucasian male samples, Caucasian female samples, German male samples, and Spanish samples, LASSO regression identified 3, 6, 3, 17, 14, 13, 23, 11, 10, 4, and 2 genes, respectively; SVM-REF identified 3, 6, 3, 6, 16, 8, 16, 6, 10, 2, and 20 genes, respectively; RF identified 3, 6, 3, 12, 5, 4, 19, 10, 12, 4, and 5 genes, respectively. Ultimately, a total of 3, 6, 3, 4, 5, 3, 11, 5, 7, 2, and 2 Hub genes were identified, respectively (Figures 5B, 6A).

In summary, DEGs were identified across various populations and genders: 3 Hub genes (MYH6, ASPN, COL14A1), 6 male-specific (NAP1L3, MOXD1, PLEKHH2, SERPINE2, CA14, CCDC80), and 3 female-specific (CX3CR1, SYN2, SLC25A18) DEGs. In addition, 4 African American-specific Hub genes (PPL, KYNU, NQO1, KAZALD1), 5 African American male-specific (NGEF, UBASH3A, HYAL4, EGR1, LRRN4CL), and 3 African American female-specific (TSPAN11, HPSE, WASF1) DEGs; 11 Caucasian-specific Hub genes (CD83, GNA14, ANTXR1, C1QTNF3, TRAF3IP3, MEG3, MARVELD2, EDN2, VIT, GRB14, CPAMD8), 5 Caucasian male-specific (DLGAP1, COL12A1, GLYATL2, GRAP2, ETNK2), and 7 Caucasian female-specific (BCL6B, CD1E. PODXL2, CERCAM, MS4A1, TIGIT, SLC5A1) DEGs; 1 German-specific Hub gene (LSAMP), 2 German male-specific (C14orf132, NECAB1), and 1 German female-specific (BMP4) DEGs; and 2 Spanish-specific Hub genes (SNORA80E, CIART). In total, 3 HF Hub genes, 18 male-specific DEGs in the African American population, 13 female-specific DEGs in the African American population, 25 male-specific DEGs in the Caucasian population, 24 female-specific DEGs in the Caucasian population, 12 male-specific DEGs in the German population, 8 female-specific DEGs in the German population, and 5 DEGs specific to the Spanish population were identified (Figure 6B).

**FIGURE 7:**
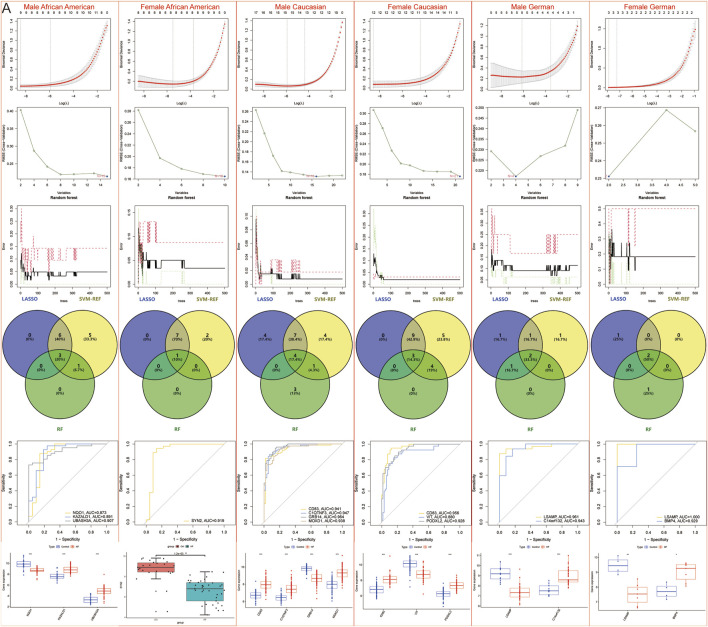
Machine learning models to screen candidate Hub genes. **(A)** LASSO, SVM-REF, and RF were again screened for Hub genes in African American male, African American female, Caucasian male, Caucasian female, German male, and German female samples.

However, to further refine the identification of specific DEGs for African American males, African American females, Caucasian males, Caucasian females, German males, and German females, machine learning techniques were again employed to reduce the risk of overfitting. LASSO identified 9, 8, 15, 12, 5, and 3 genes for each group, respectively; SVM-REF identified 15, 10, 16, 21, 4, and 2 genes for each group, respectively; RF identified 4, 1, 8, 7, 3, and 3 genes for each group, respectively. Ultimately, 3 African American males (NQO1, KAZALD1, UBASH3A), 1 African American female (SYN2), 4 Caucasian males (CD83, C1QTNF3, GRB14, MOXD1), 3 Caucasian females (CD83, VIT, PODXL2), 2 German males (LSAMP, C14orf132), and 2 German females (LSAMP, BMP4)-specific DEGs were identified (Figure 7).

### 3.4 Construction and assessment of nomogram

**FIGURE 8:**
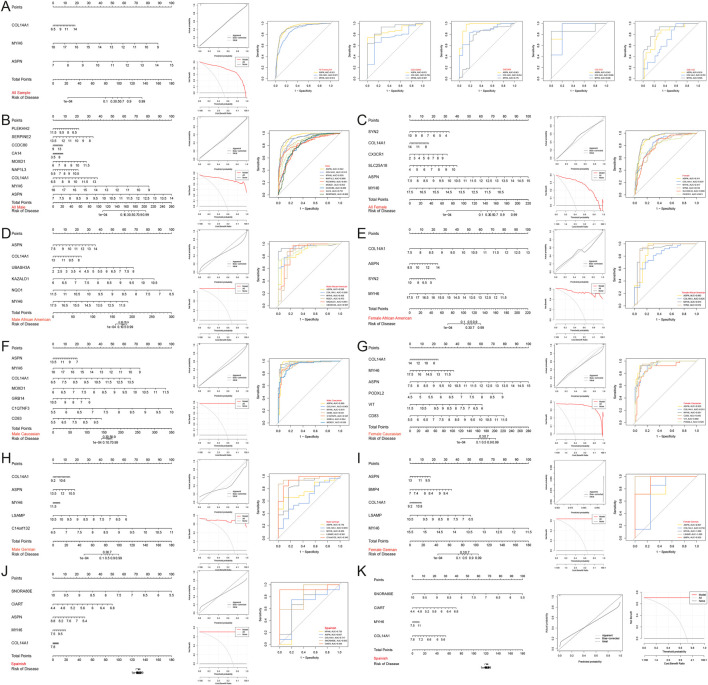
Construction and assessment of nomograms. **(A–K)** Nomograms were constructed based on the Hub gene for **(A)** all, **(B)** all male, **(C)** all female, **(D)** African American male, **(E)** African American female, **(F)** Caucasian male, **(G)** Caucasian female, **(H)** German male, **(I)** German female, and **(J,K)** Spanish samples to predict the incidence of HF; decision curves and calibration curves were used to evaluate the predictive efficiency of the models; ROC curves were used to evaluate the diagnostic efficacy of the Hub gene. AUC stands for area under the curve.

Therefore, a nomogram was constructed based on 3 Hub genes (ASPN, COL14A1, MYH6) to predict the incidence of HF in the population, and decision curve and calibration curve analyses both indicated that the nomogram effectively distinguished HF patients from the normal population (Figure 8A). Additionally, nomograms were constructed separately based on the Hub genes and population-specific DEGs for males (ASPN, AUC = 0.942; COL14A1, AUC = 0.913; MYH6, AUC = 0.935; NAP1L3, AUC = 0.880; PLEKHH2, AUC = 0.851; MOXD1, AUC = 0.853; CCDC80, AUC = 0.809; CA14, AUC = 0.791; SERPINE2, AUC = 0.803) (Figure 8B), females (ASPN, AUC = 0.911; COL14A1, AUC = 0.837; MYH6, AUC = 0.917; SYN2, AUC = 0.816; SLC25A18, AUC = 0.808; CX3CR1, AUC = 0.821) (Figure 8C), African American males (ASPN, AUC = 0.890; COL14A1, AUC = 0.930; MYH6, AUC = 0.956; NQO1, AUC = 0.873; KAZALD1, AUC = 0.891; UBASH3A, AUC = 0.907) (Figure 8D), African American females (ASPN, AUC = 0.893; COL14A1, AUC = 0.825; MYH6, AUC = 0.914; SYN2, AUC = 0.919) (Figure 8E), Caucasian males (ASPN, AUC = 0.969; COL14A1, AUC = 0.969; MYH6, AUC = 0.979; CD83, AUC = 0.941; C1QTNF3, AUC = 0.947; GRB14, AUC = 0.964; MOXD1, AUC = 0.938) (Figure 8F), Caucasian females (ASPN, AUC = 0.959; COL14A1, AUC = 0.931; MYH6, AUC = 0.951; CD83, AUC = 0.956; VIT, AUC = 0.880; PODXL2, AUC = 0.928) (Figure 8G), German males (ASPN, AUC = 0.776; COL14A1, AUC = 0.659; MYH6, AUC = 0.855; LSAMP, AUC = 0.961; C14orf132, AUC = 0.943) (Figure 8H), German females (ASPN, AUC = 0.857; COL14A1, AUC = 0.750; MYH6, AUC = 1.000; LSAMP, AUC = 1.000; BMP4, AUC = 0.929) (Figure 8I), and Spanish (MYH6, AUC = 0.733; ASPN, AUC = 0.667; COL14A1, AUC = 0.717; SNORA80E, AUC = 0.833; CIART, AUC = 0.933) (Figure 8J) to identify the incidence of HF in different regions and genders. The results similarly indicated that these nomograms effectively distinguished HF patients from the normal population. However, ROC curves for the Spanish population showed low AUC values for ASPN, and a nomogram based on the MYH6, COL14A1, SNORA80E, and CIART genes was reconstructed to predict the incidence of HF in the Spanish population. Both the decision curve and calibration curve indicated that the model effectively distinguished between HF patients and normal individuals (Figure 8K).

In addition, OMECAMTIV MECARBIL, DANICAMTIV, and MAVACAMTEN were identified through the database as potential drugs for the treatment of HF, and Collagen Alpha-1(I) Chain, Latent TGF Beta Binding Protein 2, Transforming Growth Factor-Beta Superfamily, Transforming Growth Factor Beta-1, Fibronectin, and Morpholino are common chemicals of the 3 Hub genes. Database prediction suggests OMECAMTIV MECARBIL, DANICAMTIV, and MAVACAMTEN may be associated with MYH6.

**FIGURE 9:**
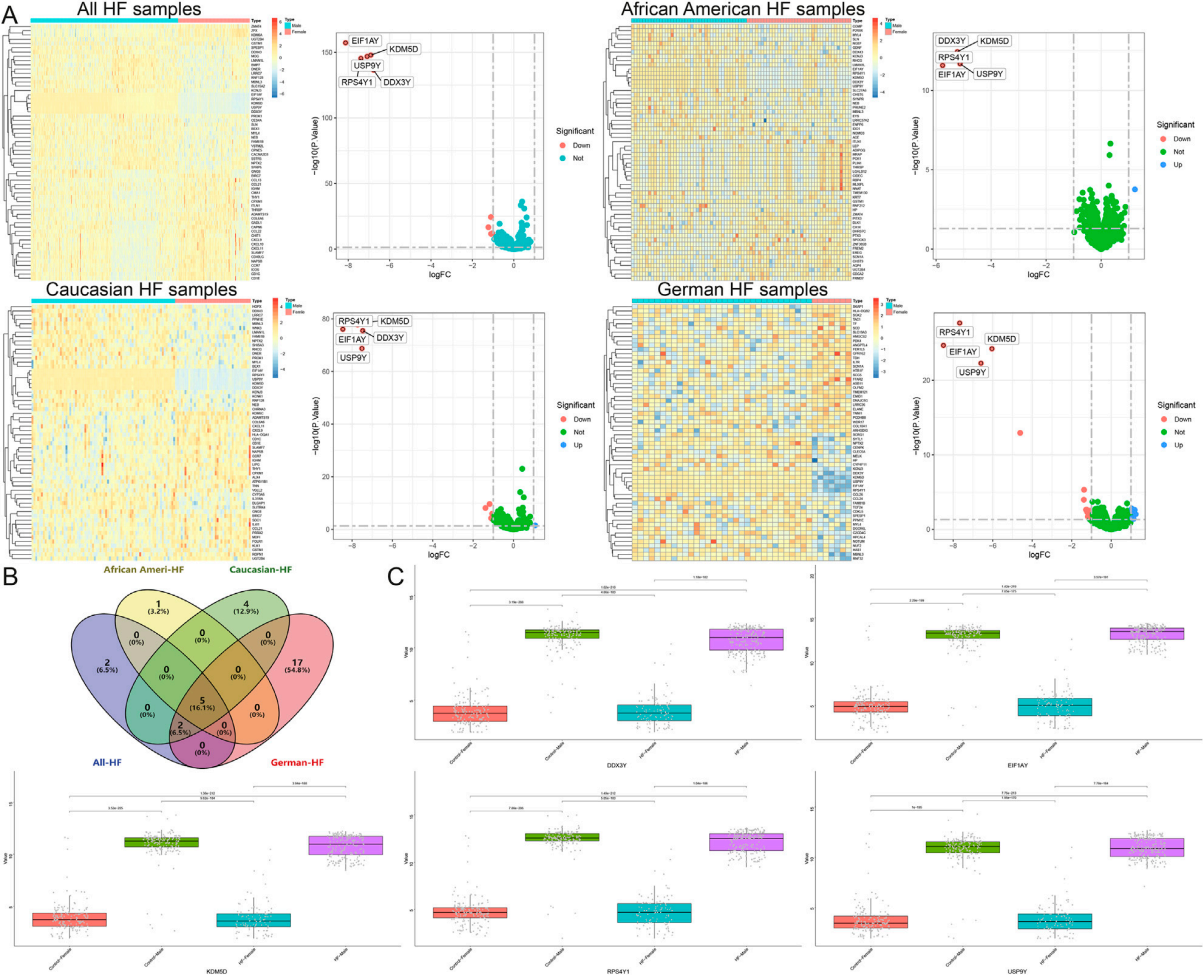
Identification of DEGs between males and females in a heart failure population from different regions. **(A)** Volcano map and heatmap for differential expression analyses of all, African American, Caucasian, and German samples. **(B)** The Venn diagram shows 5 common DEGs. **(C)** Single gene differential analysis between multiple groups of common DEGs.

Moreover, this study also stratified HF samples from different regions by gender, identifying 9 DEGs between males and females in all HF samples, 6 in African American HF samples, 11 in Caucasian HF samples, and 24 in German HF samples, and ultimately identifying 5 common DEGs that were significantly different by gender, namely, DDX3Y, KDM5D, USP9Y, RPS4Y1, and EIF1AY (Figures 9A,B). However, single-gene differential analyses across multiple groups indicated that these genes were upregulated only in female samples, both in the control and disease groups (Figure 9C).

## 4 Discussion

Symptoms, pathogenesis, and biomarkers of HF are influenced by multiple factors, particularly gender, age, and race. Several scholars have emphasized the importance of linking HF biomarkers to gender (Maidana et al., 2023; Blumer et al., 2023). Studies have shown that baseline NT-proBNP levels are higher in women than in men, especially in premenopausal women, and that other HF biomarkers, such as the soluble isoform of suppression of tumorigenesis-2 (sST2), are higher in men than in women (Maidana et al., 2023). Racial disparities also play a significant role in the development of HF. Despite improvements in HF treatments and overall survival, the mortality disparity for African American patients continues to widen over time (Lewsey and Breathett, 2021). Hale suggests that future HF research should be grounded in understanding these racial disparities (Hale and Yancy, 2023). Therefore, this study focused on race and gender to identify HF-specific biomarkers.

Enrichment analyses in this study highlight the importance of the wnt signaling pathway in the pathogenesis of HF. The Wnt signaling pathway is a fundamental cellular communication system comprising the β-linker classical pathway and the nonclassical pathways, namely, the planar cell polarity and the calcium-dependent pathways. It has been shown that the classical Wnt signaling promotes inflammation and fibrosis in the context of cardiac hypertrophy and HF (Horitani and Shiojima, 2024); non-classical WNT signaling produces contractile dysfunction by affecting myocardial oxidative stress, inflammation, reparative capacity, energetics, and remodeling, including fibrosis or fatty infiltration of the myocardium (Akoumianakis et al., 2022).

Several studies have investigated biomarkers for HF. For instance, Fan’s research identified core genes such as EIF1AY, RPS4Y1, USP9Y, KDM5D, DDX3Y, NPPA, HBB, TSIX, LOC28556, and XIST through protein-protein interaction networks (Fan and Hu, 2022). Zhu’s study identified NPPA, OMD, and PRELP as biomarkers for dilated cardiomyopathy and HF using random forests (Zhu et al., 2022). Chen’s research identified 16 differentially expressed genes (DEGs) for HF using random forests, which included ECM2, LUM, ISLR, ASPN, PTN, SFRP4, GLT8D2, FRZB, FCN3, TEAD4, NPTX2, LAD1, ALOX5AP, RNASE2, IL1RL1, and CD163 (Chen et al., 2023). Additionally, Chen identified NSG1, NPPB, PHLDA1, and SERPINE2 using LASSO and SVM-REF (Chen et al., 2022). These genes were also addressed in this study. For example, five of the ten genes identified by Fan (EIF1AY, RPS4Y1, USP9Y, KDM5D, DDX3Y) were found to be upregulated exclusively in males, in both control and disease groups; NPPA identified by Zhu did not differ in this study in the Spanish population (*P* > 0.05); Chen similarly identified ASPN as DEGs for HF; and ASERPINE2 identified by Chen was considered as male-specific DEGs in this study. In conclusion, this study encompassed datasets from multiple regions, stratified by gender, and employed three machine learning models (LASSO, SVM-REF, and RF) to identify Hub genes. First, analysis of 650 samples through differential expression analysis, DEGs, WGCNA, gender stratification, external validation across four datasets, and machine learning identified three Hub genes: MYH6, ASPN, and COL14A1.

ASPN is a member of the small leucine-rich proteoglycan family, specifically class I. Multiple bioinformatics analyses have identified ASPN as a potential biomarker for HF (Boyang et al., 2022; Guo et al., 2022; Wang et al., 2019; Huang et al., 2024). ASPN primarily encodes the asporin protein, which acts as an inhibitor of transforming growth factor-β1 (TGF-β1) and is considered a beneficial regulator of cardiac remodeling (Huang et al., 2022). In the ASPN knockout (Aspn^−/−^) mouse model, increased fibrosis and reduced cardiac function were observed following pressure overload (Huang et al., 2022). The TGFβ superfamily is one of the most important families of profibrotic cytokines in the regulation of myocardial fibrosis. Studies have shown that while inhibiting TGF-β1 may exacerbate early cardiac dysfunction, it can prevent late remodeling post-infarction, and inhibiting TGF-β1 is a significant factor in protecting the myocardium from fibrosis (Ikeuchi et al., 2004). Huang’s research found that asporin, released by cardiac fibroblasts, was able to attenuate TGFβ signaling, thereby inhibiting the progression of myocardial fibrosis (Huang et al., 2022). However, other studies have indicated that asporin plays a key role in glycated low-density lipoprotein (gly-LDL)-induced apoptosis of cardiomyocytes, increasing H9C2 cardiomyocyte apoptosis by downregulating Bcl-2, upregulating TGF-β1, Bax, type III collagen, fibronectin, and the phosphorylation of smad2 and smad3 (Li et al., 2020). On the other hand, Medzikovic’s research found that miR-129-5p expression was reduced and ASPN expression was enhanced in cardiac fibrosis and calcified human heart fibroblasts. Overexpression of miR-129-5p was able to downregulate ASPN expression, and targeting the miR-129-5p/ASPN signaling axis in cardiac fibroblasts attenuated myocardial fibrosis and calcification and restored cardiac function in mice (Medzikovic et al., 2023). In conclusion, the role of ASPN in HF is complex, but it is considered a promising potential biomarker for HF, and its specific role in HF still needs to be further investigated to clarify (Zhang et al., 2021).

COL14A1 is a major fibrillar collagen produced by fibroblasts and plays a crucial role in regulating the extracellular matrix component of the cardiac remodeling process in HF (Frangogiannis, 2019). Portokallidou has identified COL14A1 as a key gene in both dilated and ischemic cardiomyopathy through transcriptomic and proteomic analyses (Portokallidou et al., 2023). COL14A1-deficient mouse ventricles exhibit morphological defects and disorganization of collagen fibers (Tao et al., 2012). COL14A1 functions as a regulator of tissue differences, particularly during the early stages of collagen fiber formation, which is crucial for myocardial growth and structural integrity (Tao et al., 2012; Ansorge et al., 2009).

Myosin is a hexamer composed of two heavy chain subunits, two light chain subunits, and two regulatory subunits, possessing ATPase activity and the ability to bind actin. Among these components, the myosin heavy chain (MyHC) is an essential part of myocardial structure and function, playing a vital role in cardiac contractile function (Toepfer et al., 2020). The MYH6 and MYH7 genes encode the α-MyHC subunit and the β-MyHC subunit, respectively. In the human heart, α-MyHC is predominant in the atria, while β-MyHC is predominant in the ventricles (Walklate et al., 2021). The content of α-MyHC varies among different mammalian hearts: it is 100%/100% in mouse ventricles/atria, 90%/99% in rats, and 5%/75% in humans (Walklate et al., 2021). In pathological states such as HF, myosin genes may undergo “return to the fetal gene program,” where α-MyHC expression decreases and β-MyHC expression increases, leading to a reduced α-MyHC/β-MyHC ratio. This change may help maintain myocardial contractility and compensate for cardiac function in the short term, but if it persists, it can adversely affect energy metabolism (Rajabi et al., 2007; Taegtmeyer et al., 2010; Papait et al., 2013; Herron and McDonald, 2002). Several studies have shown that mRNA and protein expression levels of α-MyHC are significantly downregulated in patients with HF or cardiac hypertrophy, as well as in various animal models of HF (Reiser et al., 2001; Miyata et al., 2000; Lowes et al., 1997; Nakao et al., 1997). Furthermore, the expression of the MYH6 gene changes with the improvement of clinical symptoms during the treatment of cardiomyopathy or HF. Before treatment, MYH6 gene expression is downregulated, but it is upregulated during many therapeutic measures aimed at improving cardiac function. For instance, β-blockers can lead to upregulation of MYH6 gene expression and downregulation of MYH7 gene expression while improving ejection fraction and cardiac function. Among patients treated with β-blockers, those with improved ejection fraction exhibit an increase in α-MyHC mRNA and a decrease in β-MyHC mRNA compared to non-responders (Lowes et al., 2002).

In addition, this study identified Omecamtiv Mecarbil, Danicamtiv, and Mavacamten as potential drugs for the treatment of HF through database screening. Omecamtiv Mecarbil and Danicamtiv act as cardiac myosin activators (Nanasi et al., 2018; Kooiker et al., 2023), while Mavacamten is a cardiac-specific myosin inhibitor (Braunwald et al., 2023). Omecamtiv Mecarbil enhances myocardial contractility by specifically binding to the catalytic S1 structural domain of cardiac myosin, thereby improving cardiac function, reducing ventricular wall stress, reversing ventricular remodeling, and promoting sympathetic regression for the treatment of HF (Teerlink et al., 2020; Teerlink et al., 2021). Danicamtiv potentially enhances myocardial force and calcium sensitivity by increasing myosin recruitment and slowing cross-bridge turnover (Kooiker et al., 2023). However, Danicamtiv is still in clinical trials, and although it improves cardiac systolic function, it may limit diastolic function at high concentrations (Voors et al., 2020; Ráduly et al., 2022). Mavacamten reduces contractility by normalizing cross-bridging between myosin and actin and is commonly used to treat obstructive hypertrophic cardiomyopathy (Schenk and Fields, 2023). A meta-analysis showed that Mavacamten reduced New York Heart Association (NYHA) class and post-exercise left ventricular outflow tract gradient, and increased mixed venous oxygen pressure in patients with hypertrophic cardiomyopathy, but it may also cause adverse effects such as atrial fibrillation and reduced left ventricular ejection fraction (Bishev et al., 2023). The DGIdb database indicates that Omecamtiv Mecarbil, Danicamtiv, and Mavacamten may act on MYH6. However, there have been an absence of studies confirming the targeted effects of these drugs on MYH6, and these findings must be validated through subsequent in-depth *in vitro* and *in vivo* experiments.

This study also identified gender-specific DEGs across different regions. After stratifying by gender, differential expression analysis, WGCNA, and machine learning were performed to identify race- and gender-specific DEGs. Subsequently, a nomogram was constructed based on Hub genes (MYH6, ASPN, and COL14A1) and combined with specific DEGs to predict the risk of HF onset. Exercise stimulation induced cardiac-specific expression of the C-terminal domain of CCDC80, which prevented angiotensin II-induced cardiac hypertrophy and fibrosis in mice (Yin et al., 2022). CX3CR1 has been suggested to be a prerequisite for the development of cardiac hypertrophy and left ventricular dysfunction in mice upon transverse aortic constriction (Weisheit et al., 2021). Furthermore, the activation of the cardiac CX3CL1/CX3CR1 signaling axis delays β-adrenergic-induced HF (Flamant et al., 2021). C1QTNF3 has been found to be decreased in patients with HF with reduced ejection fraction in the Xi’an population of China and is strongly associated with increased morbidity and mortality (Gao et al., 2019). However, this study found that C1QTNF3 was upregulated in Caucasian males, which contrasts with another study that found C1QTNF3 upregulated in mouse hypertrophied hearts and in human hearts with HF, derived from cardiomyocytes and induced by the production of reactive oxygen species during the hypertrophic response. Additionally, CTRP3 facilitates pressure-overload-induced cardiac hypertrophy through activation of the TAK1-JNK axis (Ma et al., 2019). Yu identified m6A-modified C14orf132 as a potential diagnostic gene for idiopathic cardiomyopathy (Yu et al., 2024). The relationship between other genes and HF remains unclear and requires further investigation.

In conclusion, this study utilized bioinformatics to identify the pathogenesis and potential biomarkers of HF associated with dilated cardiomyopathy. First, three Hub genes (MYH6, ASPN, and COL14A1) associated with dilated cardiomyopathy-related HF were identified using a large-scale training dataset. External validation confirmed the differential expression of these genes. The research underscores that the pathogenesis of HF is closely related to inflammatory responses, immune responses, vascular regulation, the Wnt signaling pathways, glutathione metabolism, and apoptosis. The myocardial immune infiltrate microenvironment in HF patients is dysregulated and exhibits significant differences across distinct patient populations. The myocardial immune infiltrate microenvironment is dysregulated in patients with HF, characterized by a high abundance of naïve B cells and CD8 T cells, and a lower abundance of resting memory CD4 T cells, M2 macrophages, and eosinophils. Differential analyses were conducted to pinpoint population-specific DEGs, and gender-specific Hub genes were identified using three machine learning models: LASSO, SVM-REF, and RF. This study is innovative in its focus on race and gender, identifying HF-specific DEGs across different races and genders, thereby reflecting the principles of precision and individuality in medicine. Furthermore, the study identified Hub genes using multiple machine learning models, which were then validated in four external validation sets. Additionally, the study identified five genes that were upregulated only in male and not associated with HF.

However, there are limitations to this study. Although efforts were made to include HF datasets from various regions, database limitations precluded the inclusion of more HF-related datasets from other countries. The Japanese-related dataset GSE8331 was also not considered for inclusion due to its small sample size. While many specific DEGs were identified in this study, the relationship between many of these genes and HF remains unclear, necessitating further experimental validation.

However, this study has several limitations. First, the large number of datasets included in this study were sourced from various platforms and countries. Despite batch effect correction, differences in platforms, processing pipelines, and sample demographics may still introduce confounding effects that are difficult to completely eliminate. Second, although efforts were made to include HF datasets from different regions, limitations of the databases restricted the inclusion of more HF-related datasets from other countries. Moreover, there was significant variation in sample sizes across different datasets, with the Spanish cohort having a particularly small sample size, which may have reduced the reliability of the results. Additionally, while the US datasets provided detailed racial information, the population information for the other datasets was inferred based on the country of origin. However, the included populations may not have had strictly defined racial information sources, which could have affected the reliability of the results. Furthermore, although CIBERSORT has been widely applied across various tissue types, it is important to acknowledge that its reference matrix was primarily developed using peripheral blood mononuclear cells. Potential differences in gene expression profiles between blood and heart cells may have affected the accuracy of immune cell estimation. Future studies incorporating heart-specific signature matrices will help to validate and refine these findings. Age is also an important factor associated with HF. However, many of the included datasets did not provide patient age information, and thus, this study did not perform subgroup analyses based on age. This is a key aspect that needs to be addressed in future research. In addition, this study predicted drug interactions for Hub genes based on databases. However, such drug repositioning predictions are merely preliminary hypothesis-generating tools, and the reliability of their results must be verified through subsequent in-depth *in vitro* and *in vivo* experiments. Finally, although this study identified many specific DEGs, these computational results lack experimental validation. The relationships between many genes and HF remain unclear, and they have not been independently verified in patient samples or experimental models (such as qRT-PCR, Western blot, and immunohistochemistry). The biological reliability and translational applicability of the proposed biomarkers remain uncertain and require further experimental validation.

## 5 Conclusion

The biomarkers of HF vary significantly across different populations and genders. MYH6, ASPN, and COL14A1 may be potential biomarkers for HF in dilated cardiomyopathy.

## Data Availability

The original contributions presented in the study are included in the article/Supplementary Material, further inquiries can be directed to the corresponding authors.

## Abbreviations

AUC, Area Under the Curve; BNP, B-type Natriuretic Peptide; DEGs, Differentially Expressed Genes; GEO, Gene Expression Omnibus; GSEA, Gene Set Enrichment Analysis; HF, Heart Failure; LASSO, Least Absolute Shrinkage and Selection Operator; MyHC, Myosin Heavy Chain; NT-proBNP, N-terminal Pro-B-Type Natriuretic Peptide; RF, Random Forest; ROC, Receiver Operating Characteristic; SVM-REF, Support Vector Machine-Recursive Feature Elimination; TGF-β, transforming growth factor-β; WGCNA, Weighted Gene Co-expression Network Analysis.
